# A Systematic Review of the Role of Dysfunctional Wound Healing in the Pathogenesis and Treatment of Idiopathic Pulmonary Fibrosis

**DOI:** 10.3390/jcm6010002

**Published:** 2016-12-26

**Authors:** Alan Betensley, Rabab Sharif, Dimitrios Karamichos

**Affiliations:** 1 Nazih Zuhdi Transplant Institute, Integris Baptist Medical Center, Oklahoma City, OK 73112, USA; alan.betensley@integrisok.com; 2 Department of Cell Biology, University of Oklahoma Health Sciences Center, Oklahoma City, OK 73104, USA; rabab-sharif@ouhsc.edu; 3 Department of Ophthalmology/Dean McGee Eye Institute, University of Oklahoma Health Sciences Center, Oklahoma City, OK 73104, USA

**Keywords:** idiopathic pulmonary fibrosis, extracellular matrix remodeling, lung transplantation, chronic lung allograft dysfunction

## Abstract

Idiopathic pulmonary fibrosis (IPF) is a chronic, progressive interstitial lung disorder showcasing an interaction between genetic predisposition and environmental risks. This usually involves the coaction of a mixture of cell types associated with abnormal wound healing, leading to structural distortion and loss of gas exchange function. IPF bears fatal prognosis due to respiratory failure, revealing a median survival of approximately 2 to 3 years. This review showcases the ongoing progress in understanding the complex pathophysiology of IPF and it highlights the latest potential clinical treatments. In IPF, various components of the immune system, particularly clotting cascade and shortened telomeres, are highly involved in disease pathobiology and progression. This review also illustrates two US Food and Drug Administration (FDA)-approved drugs, nintedanib (OFEV, Boehringer Ingelheim, Ingelheim am Rhein, Germany) and pirfenidone (Esbriet, Roche, Basel, Switzerland), that slow IPF progression, but unfortunately neither drug can reverse the course of the disease. Although the mechanisms underlying IPF remain poorly understood, this review unveils the past and current advances that encourage the detection of new IPF pathogenic pathways and the development of effective treatment methods for the near future.

## 1. Overview of the Wound-Healing Process

Wound healing is a complex process consisting of various integrated stages, including collagen production, angiogenesis and cell migration and proliferation [1]. Coordinating these stages to ensure rapid and accurate wound repair is an essential ability for humans to sustain life. This coordination, however, is disrupted in chronic nonhealing wounds, wherein the impaired blood supply and resulting ischemia cripple cellular functions and make it difficult to deliver the basic signaling molecules [2,3]. This normal biological process takes place in four main phases: homeostasis, inflammation, proliferation, and tissue remodeling [3,4].

### 1.1. Homeostasis Phase

It is mediated by the presence of fibroblasts, new blood vessel formation, and chronic inflammatory cells (mainly macrophages) in the wound bed. The ideal wound-healing process in humans also involves an overlap of the following events: mesenchymal cell differentiation, and migration to the wound site, re-epithelialization, synthesis, and proper alignment of collagen to provide strength to the healing tissue. 

The first phase of homeostasis starts immediately after wounding, with vascular constriction and clot formation [5].This fibrin clot and surrounding wound tissue release pro-inflammatory cytokines and growth factors such as transforming growth factor-β (TGF-β), fibroblast growth factor (FGF), and platelet-derived growth factor (PDGF) [6,7].

### 1.2. Inflammatory and Cell Migration Phase

Once bleeding is controlled, chemotaxis occurs, in which inflammatory cells migrate into the wound, promoting the inflammatory phase, which is characterized by the subsequent infiltration of neutrophils and macrophages. An important function of neutrophils is the clearance of cellular debris in the wound area, although these cells also produce substances such as reactive oxygen species (ROS), which cause some additional bystander damage [8,9]. Macrophages play multiple roles in wound healing; early in the repair process, they release cytokines that stimulate the inflammatory response by recruiting additional leukocytes. Macrophages are also responsible for clearing neutrophils (apoptotic cells), thereby promoting the resolution of inflammation [3]. Macrophages then undergo a transition to a reparative state that stimulates fibroblasts, keratinocytes and angiogenesis in order to promote tissue regeneration, transitioning to the proliferative phase of healing. Although inflammation itself is essentially a protective response, persistent inflammation delays the wound-healing process, since microbial clearance is incomplete [10]. Both bacteria and endotoxins can lead to the prolonged elevation of pro-inflammatory cytokines such as interleukin-1 [11] and tumor necrosis factor alpha (TNF-α) and prolong the inflammatory phase. If this continues, the wound may enter a chronic nonhealing phase [7,10]. In addition, prolonged inflammation may also lead to an increased level of matrix metalloproteases (MMPs) [12], a family of proteases that weaken the extracellular matrix (ECM) [13]. Simultaneously, a decreased level of the naturally occurring protease inhibitors also occurs, leading to an imbalance in protease activity. 

### 1.3. Proliferation and Extracellular Matrix (ECM) Remodeling Phase

The proliferative phase generally follows, and overlaps with the inflammatory phase. It is characterized by epithelial proliferation and migration over the provisional matrix within the wound (re-epithelialization). In the reparative state, fibroblasts and endothelial cells are the most prominent cell types present and support collagen formation, capillary growth, and granulation tissue formation at the site of injury. Within the wound bed, fibroblasts produce the three major components of the ECM: collagen, glycosaminoglycans and proteoglycans. Following vigorous ECM synthesis and proliferation, wound repair enters the final remodeling state, which can last for years. In this phase, regression of many of the newly formed capillaries takes place, so that vascular density of the wound returns back to normal. One main feature of this phase is ECM remodeling to an architecture that resembles that of the normal tissue [14]. Throughout the entire healing process, the wound undergoes physical contraction which is believed to be mediated by myofibroblasts that are present in the wound bed. Various cell types are involved in the wound-healing process, and, as described above, the cellular activities of any specific cell type may also differ during different stages of wound repair [10]. Both the intricacy and coordination of the healing process are major hurdles to therapeutic approaches, considering that any therapy must effectively be matched to the appropriate wound-healing stage. 

### 1.4. Chronic Wound Healing

Various factors can interfere with one or more phases of this dynamic process, thus leading to chronic nonhealing wounds [3]. Thus, idiopathic pulmonary fibrosis (IPF) occurs as a result of constant epithelial injury leading to a highly abnormal wound-healing response in aged and genetically susceptible individuals. The typical histopathological pattern of IPF, usual interstitial pneumonia (UIP), is characterized by patchy epithelial damage, type 2 pneumocyte hyperplasia, varying degrees of fibrosis, abnormal proliferation of mesenchymal cells, and extensive deposition of collagen and various extracellular matrix (ECM) proteins. Multiple fibroblastic foci, underlying the injured epithelium represent the hallmark lesions of UIP. These lesions represent the leading edge of this fibrotic process. 

Vascular complications are often associated with chronic wounds and the resultant ischemia is one of the main contributing factors to the arrest of the wound-healing process, as the limited supply of oxygen as well as other nutrients compromises cellular functions in injury sites and interferes with tissue repair processes [5]. Therapeutic angiogenesis restores the blood supply to these ischemic wounds and promotes wound repair. Local administrations of angiogenic factors such as the Vascular Endothelial Growth Factor (VEGF) showed only limited success, suggesting that a combination of cytokines and growth factors is needed to acquire neoangiogenesis with functional vasculatures [5,6]. 

### 1.5. Etiology

Lung wound repair is a complicated, highly orchestrated process presenting various events where dysregulation may occur, leading to the development of several pulmonary disorders. Current studies are limited by a lack of relevant lung injury models. Lungs have a substantial potential for recovery after lung injury. Thus, lung wound repair is critical for restoration of lung homeostasis. The initial events following injury include an acute inflammatory response, leading to immune cell recruitment, and epithelial cell migration upon an autologously secreted temporary matrix. Injury causes the release of factors that contribute to repair mechanisms including members of the epidermal growth factor (EGF) and fibroblast growth factor (FGF) families [15,16]. However, the mechanisms that cause tissue disruption in the early phase can also facilitate its repair later on, inflammation and matrix remodeling being paradigmatic examples. Therefore, therapies that disrupt these pathways, such as matrix metalloproteinase (MMP) inhibition, may have a prophylactic value, but their application at a later phase could be harmful. 

Knowledge of the mediators involved in tissue repair could lead to new therapeutic strategies being applied after the initial insult has been controlled. Tissue fibrosis, a considerable cause of death worldwide, advances to obvious organ dysfunction of the human body. Lung fibrosis severely impairs tissue oxygenation, such as in the case of IPF which is the most lethal fibrotic disease of the lung, with a predicted median survival of only 3–5 years [17]. The only way to replace damaged lung with healthy in IPF is lung transplant. However, lung function post-transplant is compromised by severe complications, predominantly chronic lung allograft dysfunction (CLAD). Surprisingly, the mechanisms leading to fibrosis in the engrafted lung resembles those in IPF; therefore, antifibrotic treatments may contribute to improved graft function in CLAD [18]. In this review, we focus on the fibrosis-mediated mechanisms in IPF, analyzing clinical phenotypes, biomarkers, and mechanisms of fibrogenesis, in order to predict disease status.

## 2. Overview of Idiopathic Pulmonary Fibrosis (IPF)

### 2.1. Prevalence/Incidence

IPF is a progressive, fibrotic lung disease that typically develops in the 6th or 7th decades of life [19]. More males have been reported with IPF than females [20]. At the time of disease presentation, roughly two-thirds of patients with IPF are over the age of 60 years, with a mean age at diagnosis of 66 years [21]. Table 1 outlines the prevalence and incidence estimates per 100,000 populations by geographic district [22,23]. The incidence of the disease increases with older age [19,20]. In one study, incident cases tended to be older than prevalent cases [24]. Prevalence for adults 35 to 44 years was 2.7 per 100,000; by contrast, prevalence exceeded 175 per 100,000 for individuals older than 75 years [25].

### 2.2. Treatment Guidelines 

IPF was once thought to be driven by inflammation, and older studies such as the 2000 consensus statement have looked at treatment with agents such as prednisone, azathioprine, and cyclophosphamide [35,36]. These guidelines have unfortunately followed unstructured approaches and have made inappropriately strong recommendations without considering the certainty in the evidence. More recently, anti-fibrotic therapy has been proven effective, and anti-inflammatory therapy may even be considered harmful. Nevertheless, IPF treatment options need to fulfill two main requirements—safety and efficacy—which can affect the course of IPF through slowing disease progression. Recently, the 2015 international evidence-based IPF guideline, developed by a joint international committee including the American Thoracic Society (ATS), the European Respiratory Society (ERS), the Japanese Respiratory Society (JRS) and the Latin American Thoracic Association (ALAT), supplied physicians with updated treatment recommendations for IPF patients. These updated recommendations were formulated using the common terminology of “we suggest” for weak recommendations and “we recommend” for strong recommendations. This evidence-based approach aids clinicians to provide a more personalized treatment strategy when treating patients with IPF [37].

### 2.3. Diagnosis

Pathology of IPF is generally derived from the usual interstitial pneumonia, where the fibrotic areas consist mainly of dense type V collagen, and scattered foci of proliferating fibroblasts (fibroblastic foci) are also present [17]. UIP can also be seen in other disorders such as collagen vascular disease, occupational/environmental lung disease, and chronic hypersensitivity pneumonitis [38]. However, in collagen vascular disease, the most common pattern of lung injury is nonspecific interstitial pneumonia (NSIP), which can be primarily inflammatory (cellular NSIP) or (fibrotic NSIP). [39,40]. Almost all IPF patients have an anomalous chest radiograph at the time of presentation [41]. In individuals with such asymptomatic abnormalities, investigation by physiological evaluation or by high-resolution CT (HRCT) scanning could facilitate earlier detection and treatment of IPF. A characteristic HRCT in many cases can be diagnostic for IPF, eliminating the need for lung biopsy. Conversely, a normal chest radiograph cannot be used to exclude microscopic evidence of UIP on lung biopsy [42].

In the proper clinical scenario, pulmonary function testing may reveal low forced vital capacity (FVC), total lung capacity (TLC), and forced expiratory volume in one second (FEV1) with elevated FEV1/FVC ratio, along with clinical finding of dyspnea and hypoxemia in a patient in the sixth or seventh decade [17]. Despite the fact that cell count of bronchoalveolar lavage (BAL) fluid from patients with IPF can have a high differential distribution of eosinophils or lymphocytes, the role of BAL in IPF diagnosis remains limited [38]. Trans-bronchial biopsy (TBLBx) is likely to be non-diagnostic when advanced fibrotic disease is present. However, a surgical lung biopsy (SLB) acquired via open biopsy or video-assisted thoracic surgery (VATS) is likely to provide an adequate specimen that shows a definitive diagnostic histopathological pattern of a specific disease entity [43]. Nevertheless, one must weigh risks and benefits when performing an SLB, especially in ventilator-compromised elderly patients, or patients with multiple co-morbidities, such as severe pulmonary hypertension [17]. Following proper diagnosis of IPF, first-line treatment includes supplemental oxygen, pulmonary rehabilitation, and referral to a lung transplant center. Due to the potential for rapid progression of disease, delaying referral can have grave consequences. In 2014, the FDA approved two new drugs, pirfenidone (Esbriet, InterMune, Inc., Brisbane, CA, USA) [44,45] and nintedanib (OFEV, Boehringer Ingelheim, Germany) [46] which will be described in greater detail later in this review.

## 3. Potential Treatment, Targets of Intervention

### 3.1. Non-Pharmacologic Management

#### 3.1.1. Home Pulse Oximetry

As pulmonary fibrosis progresses, normal lung is replaced by scar tissue. This impairs the lung’s ability to exchange gas and deliver oxygen into the blood, resulting in hypoxia [47]. Home pulse oximetry provides a noninvasive assessment of arterial oxygen saturation of hemoglobin (S_p_O_2_) by measuring light-absorbing variations resulting from arterial blood flow pulsations. Home pulse oximetry is used in IPF patients to monitor and maintain S_p_O_2_ at a level above 90% to prevent the development of secondary pulmonary hypertension [48].

#### 3.1.2. Pulmonary Rehabilitation

Based on two controlled trials of pulmonary rehabilitation showing an improvement in walk distance and symptoms or quality of life, it has been suggested by the ATS, ERS, JRS, and ALAT panel of experts as a treatment option to reduce dyspnea severity, improve exercise capacity, and improve well-being of IPF patients. These programs are mostly out-patient-based, and run by a team including doctors, physiotherapists and other healthcare specialists. However, they did suggest that not using pulmonary rehabilitation could be considered in some patients with IPF.

Therefore it has been noted that exercise programs should be adapted to the patient’s daily life so as to maintain the gained positive effects of the pulmonary rehabilitation program, and home-based programs may be a suitable option [49].

#### 3.1.3. Lung Transplantation

Due to potential rapid progression of disease, a limited window of opportunity may exist to refer IPF patients for lung transplant evaluation. The expected median survival is 34 months after the initial diagnosis of IPF, but the window of opportunity for lung transplant can be much shorter. The timing of referral is dependent on the expected wait time, which can vary greatly depending on the individual transplant center. In addition, many transplant programs require completion of at least 3 months of pulmonary rehabilitation before being eligible for transplant. Because it is impossible to predict the rate of progression of disease in a patient with IPF, it is recommended to refer to a transplant center for initial evaluation at the time of diagnosis [50]. Pulmonary function testing (PFT), as a noninvasive quantitative measurement, is the keystone of current practice in the assessment of the disease progression and severity [48,51]. IPF has become the most common diagnosis for lung transplant, now representing over 50% of lung transplants in the United States [52]. Many transplant programs will consider accepting IPF patients up to 75 years of age. Although lung transplant can improve the quality of life as well as improve survival for individuals with IPF, complications after lung transplants are still more common than that after heart, kidney, or liver transplant. A 5-year survival post lung transplant is slightly greater than 50% according to the International Society of Heart and Lung Transplant (ISHLT) registry [52,53].

### 3.2. Pharmacologic Management

The pharmacological treatment of IPF has changed dramatically in the last decade, reflecting the evolving understanding of IPF pathogenesis. Initially, the general hypothesis was that a persistent inflammation ultimately triggered scarring of the lung. As such, primitive studies evaluated the potential efficacy of drugs that primarily halt immune and inflammatory responses, such as corticosteroids and immunomodulatory agents; the results of these trials have all been uniformly disappointing [54,55]. Over the last decade, the outlook on pulmonary fibrosis pathobiology has profoundly changed, and current concepts suggest that there is an initial alveolar epithelial cell damage followed by an unusual healing response resulting in the migration, activation and proliferation of mesenchymal cells, accompanied by focal accumulation of myofibroblasts, known as fibroblast foci. Progressive buildup of ECM proteins and destruction of lung architecture complete the histopathological picture [14]. Accordingly, more recent randomized controlled trials have shifted their focus to molecules with anti-proliferative and anti-fibrotic properties [54,56]. However, the pathogenesis of IPF remains incompletely understood. Several drugs approved for the treatment of other diseases, but with some evidence of potential efficacy in fibrotic disorders, have also been evaluated in IPF clinical trials. Here we discuss the most widely used pharmacological treatment of IPF. Two recent trials have proved that the combination treatment of azathioprine, prednisone, and *N*-acetylcysteine (NAC) is ineffective in IPF patients [57,58]. In contrast, several recent trials have proven that pirfenidone and nintedanib can be effective in prohibiting the decline in lung function in IPF patients. 

#### 3.2.1. Corticosteroids

On the basis of early observations demonstrating inflammatory cells in the distal air space, multiple studies have investigated the use of corticosteroids in IPF. Some early studies suggested that corticosteroids reduced the so-called “ground-glass opacities” of HRCT in patients with interstitial pneumonia, and this reduction mirrored improvement in pulmonary function; however, the progression to irreversible honeycomb fibrosis is not altered [59]. At present, no evidence for an effect of corticosteroids in IPF is available. Considering the massive clinical experience and developments in the understanding of the pathogenesis of IPF, it is reasonable to assume that appropriate trials to investigate the efficacy of steroids in IPF will never be carried out. Alternatively, a trial of steroids should be considered for those patients with idiopathic interstitial pneumonia but without a definite diagnosis of IPF, when another diagnosis cannot be ruled out. In such cases, a trial of prednisone should be limited to 3–6 months, due to the fact that longer trials of prednisone and the prolonged use of cytotoxic agents remain controversial. In the absence of measurable improvement, steroid therapy should, in most instances, be discontinued [60].

#### 3.2.2. Anticoagulation Trials in IPF

Studies in experimental animal models have provided an important case that blocking the coagulation cascade and, precisely, the pro-fibrotic effects of coagulation proteinases, may hold promise for the treatment of IPF [61]. In terms of long-term anticoagulant therapy, it is now increasingly recognized that therapeutic approaches based on selective inhibitors of coagulation factors, such as FXa, rather than classical, multi-targeted anticoagulants, such as warfarin and unfractionated heparin, are likely to be associated with a wider therapeutic window and therefore be safer to use [62]. Despite the success of anticoagulant strategies in experimental models of lung injury, clinical trials to date have proven unsuccessful in improving patient outcomes In IPF, although a small clinical study of anticoagulant therapy in IPF provided some initial supportive evidence for the usefulness of anticoagulants in this disease setting, this phase III multicenter ACE-IPF (Anticoagulant Effectiveness in Idiopathic Pulmonary Fibrosis) trial was terminated early for ineffectiveness and excess mortality in the warfarin treatment group [63,64]. These trial data are disappointing, but it is worth bearing in mind that coagulation exerts highly complex, multifaceted effects on inflammation, hemostasis, and tissue repair. Treatment strategies that target detrimental procoagulant, and profibrotic responses without compromising tissue repair are likely required in these disease settings. Strategies aimed at specifically targeting coagulation cascade responses within the intraalveolar compartment may also provide advantages over traditional anticoagulants. In this regard, nebulized administration of anticoagulants might represent a means of achieving local anticoagulation without undesired systemic effects. There is vital epidemiological evidence of an association between IPF and thrombotic vascular events, supported by biological display of a local and systemic prothrombotic state, which interacts with disease severity and clinical outcomes [61]. Further research is required to define the mechanisms underlying the prothrombotic state in IPF, to assess the utility of phenotyping patients based on thrombotic tendency and to study new classes of anticoagulants in IPF.

#### 3.2.3. *N*-Aetylcysteine

Previous assumptions show that an oxidant-antioxidant imbalance may contribute to the pathogenesis of IPF. *N*-acetylcysteine (NAC), a precursor of the endogenous antioxidant glutathione [65], has been used in IPF. Previous studies failed to meet the endpoint of a significant change in FVC in patients with early disease. Nevertheless, a study that was part of the IPF net-sponsored PANTHER trial had been originally designed to compare three therapeutic interventions, azathioprine, prednisone, and high-dose NAC (600 mg three times daily), NAC alone, or placebo [54]. However, this combination therapy regimen was terminated due to major safety issues, and the study continued as a two-arm study only (NAC monotherapy versus placebo). In comparison with placebo, NAC showed no significant effect in terms of preservation of FVC in patients with IPF [54,57]. Evidence-based guidelines recommend that NAC therapy may be a reasonable choice in a minority of patients, and the majority of patients with IPF should not be treated with *N*-acetylcysteine monotherapy.

#### 3.2.4. Nintedanib

##### Mode of Action

Nintedanib, originally developed as an anticancer drug, is an orally administered inhibitor of multiple tyrosine kinases that targets vascular endothelial growth factor receptors (VEGFR) 1–3, fibroblast growth factor receptors (FGFR) 1–3, and platelet-derived growth factor receptors (PDGFR). VEGF, FGF, and PDGF connect a number of processes including angiogenesis and fibrogenesis and have been implicated in the pathogenesis of IPF [66].

##### Clinical Efficacy

The efficacy of nintedanib in pulmonary fibrosis patients has been evaluated in two identical, randomized, double-blind, placebo-controlled, 52-week duration, phase III trials (INPULSIS-1 and INPULSIS-2). A total of 1066 patients were randomly assigned in a 3:2 ratio to receive nintedanib 150 mg twice daily [67]. In both trials nintedanib significantly reduced the rate of decline in forced vital capacity (FVC) over the study period. In addition, in both trials, patients in the nintedanib treatment arm were significantly more likely than patients in the placebo arm to be functionally stable at week 52 [68].

##### Adverse Effects

Overall, nintedanib showed an acceptable safety profile as shown by the comparable incidences of adverse events across all groups. The most common drug-related side effects were nausea, diarrhea, and vomiting [69].

#### 3.2.5. Pirfenidone

##### Mode of Action and In Vivo Activity

Pirfenidone is a synthetic pyridone compound, available orally. Although its mechanism of action is not fully understood, animal model studies of pulmonary fibrosis suggest that it exerts antifibrotic and anti-inflammatory properties through inhibition of cytokines and inflammatory cells, and the downregulation of key pro-fibrotic growth factors, including transforming growth factor-β (TGF-β). Antioxidant activity was also demonstrated in vitro at rather artificially high concentrations [69,70,71]. 

##### Clinical Efficacy

Moreover, a specified analysis including data from two CAPACITY studies showed that pirfenidone compared with placebo significantly reduced both all-cause mortality and IPF-related mortality at 52 weeks. 

##### Approval

In order to approve the usage of pirfenidone in the USA, the US Food and Drug Administration (FDA) required an additional randomized, placebo-controlled, double-blind trial called ASCEND, to confirm the effect of this drug on disease progression. Compared with the placebo, pirfenidone reduced the decline of FVC at week 52 and the relative risk of death by 43%. Following trial results, pirfenidone safety has been demonstrated in both CAPACITY and ASCEND studies. 

##### Adverse Effects

Commonly reported treatment-related adverse events included nausea, dizziness, vomiting, photosensitivity reaction, insomnia, and abdominal distension, which however were mild to moderate in severity and reversible and without clinically significant events [69]. The 2015 guidelines give a conditional recommendation for use of pirfenidone in patients with IPF (e.g., “this recommendation puts a high value on the potential benefit of pirfenidone on patient-important outcomes such as disease progression as measured by rate of FVC decline and mortality and a lower value on potentially significant adverse effects and the cost of treatment”) [72].

#### 3.2.6. Supplemental Oxygen Therapy

Hypoxemia is common in patients with IPF, impacting their quality of life and leading to the development of pulmonary hypertension if left untreated. Daytime oxygen concentration is the best predictor of nocturnal hypoxemia [73]. Nocturnal hypoxemia is associated with reduced energy levels and impaired daytime physical and social functioning. Long-term oxygen therapy (LTOT) is strongly recommended in patients with IPF and resting hypoxemia, although the evidence is derived from the study of chronic obstructive pulmonary disease (COPD). A panel of experts from the ATS, ERS, JRS, and ALAT believed that, in addition to extrapolation of COPD data, the physiological rationale and the ethical concern over withholding supplemental oxygen in a patient with resting hypoxemia strongly supported LTOT in IPF.

Supplemental oxygen has also been used during rehabilitative exercise training. Patients should be reassessed regularly and oxygen prescriptions should be altered as oxygen demands change [74]. In obstructive lung diseases, exercise capacity is limited due to ventilatory insufficiency, impaired respiratory mechanics, and musculoskeletal dysfunction, whereas the most important factor limiting exercise capacity in IPF is circulatory impairment that causes exercise induced gas exchange impairment [75]. The exercise-induced hypoxemia leads to inactivity during daily life with the disease progression causing pathophysiological impairments and decreases in health-related quality of life in these patients. 

### 3.3. Lung Injury/Epithelial Cell Death

Repeated injury to the alveolar epithelium, accompanied by the loss of epithelial cells and abnormal tissue repair, is exhibited in IPF. This is characterized by deposition of ECM factors, excessive accumulation of fibroblasts and myofibroblasts, and malformation of lung architecture, eventually leading to respiratory failure [17,47]. Fibroblast activation and accumulation in IPF, however, appears to be fundamentally driven by chronic, non-resolving injury to the alveolar epithelium [76]. Thus, the injured alveolar epithelium can be thought of as the causative edge of active fibrosis. With fibroblasts and alveolar epithelial cells being in close apposition in the lung, it is not unusual that the synergy between these two key cellular members contribute to the development of pulmonary fibrosis [77].

#### 3.3.1. The Adult Lung Epithelium

The epithelium within each region of the conducting airway is composed of specific types of epithelial cells. The integrity of epithelium is essential for maintaining normal lung functions. In adults, type 1 and 2 alveolar cells compose the epithelial component of the alveoli [78]. Alveolar type 2 cells are multifunctional in that they synthesize and secrete pulmonary surfactant, serve as progenitor cells for type 1 alveolar cells, and contribute to the immune response by producing molecules involved in innate host defense. Other lung epithelial cell types include: secretory club and goblet cells, ciliated cells, neuroendocrine cells, and basal cell types that shape the tracheo-bronchial pseudostratified epithelium. Both secretory and ciliated cells work together to clear pathogens and other debris from the airways [79]. Mucous and goblet cells produce and release mucous into the apical surface of the epithelium thus trapping foreign particles which are then cleared out by the action of ciliated cells flowing in a rhythmic movement. Mucous is composed mainly of mucins, which are highly charged glycoproteins, and some anti-viral and anti-inflammatory components such as lysozyme, IgA, and various cytokines. Mucin production becomes up-regulated following viral infection to permit better trapping and elimination of viral particles. However, over-production of mucous can have detrimental effects by creating mucous plugs and thus leading to airway obstruction, which is a typical feature of several chronic lung diseases such as chronic obstructive pulmonary disease (COPD), asthma, and cystic fibrosis [80]. Nevertheless, epithelial permeability and integrity are maintained by tight communications within the epithelial cell layer, such as adherens junctions, tight junctions and desmosomes. These cell–cell junctions permit strong adhesion, retain mechanical strength in the tissue, allowing communication between neighboring lung cells and preventing the entry of inhaled allergens, bacteria, and viruses into the basolateral membrane where they can approach epithelial cell receptors and activate antigen-presenting cells (APC) [77]. 

#### 3.3.2. The Injured Epithelium and IPF

An escalation in the number of epithelial cells and a significant disturbance in the integrity of the alveolar epithelium with the presence of several altered phenotypes is a noteworthy feature of IPF lungs. Constant damage to the epithelium and accompanying cell apoptosis are thought to contribute to the constancy of the fibrotic scarring [81]. Although the causative act that initiates the fibrotic deluge in IPF is still unidentified, apoptosis of epithelial cells is considered the main initiator event. Cell senescence due to genetic factors may trigger the apoptotic cascade in epithelial cells, as well as environmental factors; viral infections, cigarette smoking, and gastroesophageal reflux (GER) are a few of the possible causative factors [82]. Genetic mutations of an enzyme that adds telomere repeats to the end of linear chromosomes, known as telomerase, may occur in 10% of familial IPF. This enzyme is known to sustain the precursor function in type II alveolar cells and enzyme alteration significantly affects their regenerative capacity. Shortening of the telomere is alarming for the cell as it causes DNA damage and promotes cell death [83]. In addition, other disease-linked mutation may also lead to alveolar epithelial cell apoptosis. Surfactant protein C gene mutations found in familial IPF result in abnormal surfactant protein folding and accumulation in the cell cytoplasm. In an attempt to recover cell death, an unfolded protein response (UPR) is activated, which halts protein production. If this mechanism is not cleared up, the cell enters a state of distress, called endoplasmic reticulum (ER) stress, and eventually leads to cell apoptosis. Environmental factors, like viral infections, and cigarette smoking can induce UPR and ER stress, and contribute to speeding up telomere shortening and cellular senescence in the lung epithelia. In addition to above-cited environmental and genetic factors that possibly induce epithelial cell injury in lung fibrosis and the various mechanisms it activates, which perpetuate both damage and repair responses [84], mechanical stress to the lung is an additional cofactor in promoting alveolar damage. The mechanical stretch fixated on specific parts of lung parenchyma has distinct effects on tissue regeneration, and alveolar epithelial permeability following injury. Thus, various studies have shown that inducing mechanical stretch in in vitro cultures of epithelial cells inhibited wound closure through inhibiting cell migration. There is significant evidence that IPF is molded by complex interaction between constant epithelial injury, aging process, and genetic susceptibility. However, there is also evidence that the TGF-β pathway and distorted ECM are important players in initiating the fibrogenic response via various feed-forward loops. Therefore, continuous fibrosis may symbolize the extremes of a severely dysregulated tissue injury response [85].

### 3.4. Clotting/Coagulation

The normal response of tissue to injury requires a sequence of coincidental events, which need to occur in a time-controlled manner for successful tissue repair and recovery of normal function. Failure to control the healing process can lead to extensive tissue remodeling and the replacement of functional tissue with permanent fibrous scar tissue. One of the main events initiated following tissue injury is the activation of the coagulation cascade. The role of the coagulation cascade extends beyond its role in hemostasis in that coagulation proteinases influence a number of cellular responses which are critical to wound healing [63]. In the event that coagulation signaling is dysregulated, these cellular responses may then contribute to the tissue fibrosis. In the following sections, this article will focus on the role of coagulation signaling in relation to pulmonary fibrosis. 

#### Coagulation in IPF

One of the fundamental events following tissue injury is the activation of the coagulation cascade. Activation of the coagulation pathway is a feature of a number of lung disorders associated with excessive deposition of ECM, including IPF [61].

It is known that the importance of the coagulation cascade extends beyond its role in hemostasis and that coagulation proteinases influence a number of cellular responses which are critical to wound healing. If the coagulation signaling is dysregulated, these cellular responses eventually lead to the tissue fibrosis. One of the main functions of this cascade is the formation of insoluble cross-linked fibrin strands that bind and stabilize weak platelet hemostatic plugs formed at sites of tissue injury. The formation of this interim clot is vitally dependent on the action of thrombin, and is generated following the activation of coagulation proteinases via the extrinsic and intrinsic systems [61,86].

Previous studies indicate that a tissue factor-dependent extrinsic coagulation pathway is the prevailing mechanism by which the coagulation cascade is locally activated in the lungs of patients with IPF. Tissue factor (TF) is highly upregulated in type II pneumocytes, and to some extent on alveolar macrophages, and is in close association with fibrin deposits in the lungs of patients with pulmonary fibrosis. TF-positive cells are found overlying fibrotic areas, suggesting that local TF expression and extravascular activation of the coagulation cascade is closely related to fibrin deposition in IPF [63].

The intrinsic pathway is under the influence of antithrombin which inhibits thrombin and other serine proteinases. One main mechanism involves the binding of thrombin to the endothelial cell surface receptor, thrombomodulin. During this event, thrombin is converted from a pro-coagulant into an anticoagulant by activating protein C. There is good evidence that in patients with pulmonary fibrosis, the balance is greatly shifted in favor of both antifibrinolytic and pro-coagulant activity [86]. The main effector enzyme in the fibrinolytic system is plasmin, which is derived from plasminogen via the action of urokinase-type plasminogen activator and tissue-type plasminogen activator, produced by endothelial cells, alveolar epithelial cells and macrophages. Studies in patients with pulmonary fibrosis have shown that inhibitors of this pathway, including plasminogen activator inhibitor-1 (PAI1) and PAI2, thrombin-active fibrinolysis inhibitor, and protein C inhibitor are increased in the lungs of patients with pulmonary fibrosis. Decreased protein C activation was further found to be associated with abnormal collagen turnover in the intra-alveolar space in patients with interstitial lung disease [87].

### 3.5. Immune Activation

The primary mechanism underlying IPF had long been considered to be a chronic inflammatory process. However, current theory suggests that IPF results not from inflammation but rather from a fibrotic process [82]. In addition, fibrogenesis is believed to result from constant injury of alveolar epithelial cells followed by dysfunctional repair. Recently, the inflammatory process has been described as mild, consisting of a scanty interstitial infiltrate of lymphocytes and plasma cells, and is not considered as a critical factor related to IPF pathology [88]. Since the injury to the epithelium most likely triggers an immune response in the lung, the coagulation pathway is the primary response mechanism activated in the wound-healing process, and stimulated platelets release pro-fibrotic factors like PDGF and TGF-β1. Impaired epithelial cells release several chemokines that recruit inflammatory cells such as monocytes and neutrophils to the site of injury [1,8]. In a specific injury, such as infection, monocytes differentiate into phagocytic macrophages that phagocytose the fibrin clot, while neutrophils remove debris and destroy invading bacteria. IPF is considered a case of repeated injury in which neutrophils and macrophage elimination is slow as compared to normal tissue. Therefore, their presence can further aggravate the fibrotic process by continuous ROS production [89]. An early predictor of high IPF mortality in patients is the signaling of neutrophils to the bronchoalveolar space, as well as recruiting other innate myeloid cell types that have a pro-fibrotic role in the lung including eosinophils, mast cells and innate lymphoid cells 2 (ILC2), as seen in hepatic fibrosis cases. In previous research, evidence reveals that CD4+ Th1, Th2, and Th17 subtypes may have a functional role in lung fibrosis. Regulatory T cells have also been correlated with pulmonary fibrosis; however, there is still some argument as to whether their participation in IPF is pro- or anti-fibrotic [90].

In addition, toll-like receptor (TLR) activation in epithelial cells could be responsible for the epithelial-induced immune cell response in the lungs, these receptors are also linked to tissue repair, through enhancing tissue remodeling. TLR9 has been overexpressed in fibroblast of IPF patients [82], as well as TLR2, that was also found up-regulated in pulmonary tissue of IPF patients when compared with healthy controls. This receptor has shown to be critical for the release of pro-inflammatory cytokines and the activation of collagen and fibronectin deposition post bleomycin exposure [76]. In a radiation-induced lung fibrosis model [91], both TLR2, 4 were found to possess protective effects via preventing epithelial cell injury and inhibiting fibrogenesis. In recent studies, TLR3 is portrayed as an important player in IPF, being that in IPF-derived fibroblasts with TLR3 L412F polymorphism, TLR3 activation leads to abnormal cytokine production. Moreover, in animal studies, TLR3-knockout mice reveal increased collagen production following bleomycin-induced fibrosis in IPF patients [92]. This revealed greater risk of mortality, as well as a rapid decline in forced vital capacity. It is also known that TLRs have shown to identify some endogenous ligands, including specific forms of hyaluronic acid, which concludes a major component of the ECM. More studies show that, fibrinogen cleavage products also act as TLR4 ligands in both epithelial cells, and alveolar macrophages, and that this connection up-regulates the gene expression of IL-13Rα1 and MUC5AC in both these cell types. These endogenous patterns are described as danger-associated molecular patterns (DAMPs) and it states importance in chronic lung diseases, such as IPF [93]. Therefore, in patients with IPF, the prevalence of data suggests that alveolar macrophages (AM) demonstrate an alternatively activated phenotype. In comparison between IPF patients and healthy controls, AM showed, and spontaneously generated, higher levels of the proinflammatory cytokines CCL17, CCL18, and CCL22, and highly expressed CD206 [94]. 

#### 3.5.1. Fibroblast Accumulation/Myofibroblast Differentiation

Epithelial injury and dysfunctional epithelial regeneration are the main events involved in initiating and maintaining IPF. However, current evidence points to fibroblasts and highly contractile myofibroblasts as the key player cells accountable for disproportionate matrix synthesis and deposition in pulmonary fibrosis [95]. Identifying the cellular origin of these myofibroblasts in tissue fibrosis is still an area of ongoing research debate. In general, a significant portion of myofibroblasts in IPF are likely derived from the proliferation, recruitment, and differentiation of resident lung fibroblasts. Several animal model studies have identified additional cellular sources, including epithelial cells (via EMT), circulating bone marrow-derived fibrocytes, endothelial cells (via endothelial to mesenchymal transition), and pleural mesothelial cells (via mesothelial to mesenchymal transition) [77,82]. Indeed, in terms of the influence of EMT on the fibrotic response, there is an increasing appreciation that epithelial cells undergoing EMT process are likely to contribute to the aberrant epithelial-mesenchymal crosstalk that enhances fibrogenesis, rather than act as a significant source of myofibroblasts. 

#### 3.5.2. Role of Epigenetics in IPF Fibroblast/Myofibroblast Activity

Recently, it has been recognized that epigenetic influences promote the regulation of gene expression, as an important mechanism by which multiple factors including environmental stressors, such as tobacco smoke and aging, can promote changes in the phenotype of a cell or tissue. These epigenetic changes include: various alterations in DNA methylation, microRNA expression, or histone modification. Changes in DNA methylation coincide with mRNA expression for multiple genes, playing critical roles in IPF, such as specific regulation of apoptotic cellular processes [96]. These findings predict that epigenetic changes are important in IPF pathobiology, and that their possible influences on gene expression may explain the unstoppable progression of this fatal illness. Several studies showed that DNA methylation analysis studies comparing different levels of 5-methyl-cytosine (5-mC) in IPF and normal lung tissue samples have revealed significant alterations in the CpG island methylation profile between both groups. In comparison with normal lung tissue, IPF samples displayed global hypomethylation. Other studies quantified DNA methyltransferase (DNMTs) expression in IPF tissue, showing an increased mRNA expression level of de novo enzymes and increased DNMT3a protein expression in the diseased lung [97]. Methylation profile of specific cell types is concealed when whole tissue analysis is performed, indicating that fibroblast studies have a hyper-methylated profile in these cells, promoting myofibroblast differentiation, leading to disease progression. Enhanced DNA methylation level leads to gene silencing through several mechanisms. First, high levels of 5-mC can directly prohibit transcription factor binding, thus inhibiting gene transcription. Second, methylated DNA activates methyl binding domain proteins (MBDs), which typically lead to chromatin remodeling via histone-modifying enzymes. Expression of a specific MBD protein, *MeCP2*, participates in promoting gene silencing. Inhibition of *MeCP2* expression in lung fibroblasts increases alpha smooth muscle actin (αSMA) expression, a classical feature of myofibroblast differentiation. Furthermore, in various studies incorporating the bleomycin-induced lung fibrosis model, *MeCP2*^−/−^ displayed less myofibroblast differentiation, lung tissue collagen deposition and decreased myofibroblast differentiation compared with wild-type animal models [65]. Nevertheless, *MeCP2* can potentially bind to both methylated and unmethylated DNA, in order to assist and promote chromatin remodeling. Therefore, several studies linking *MeCP2* to myofibroblast differentiation all reveal changes in DNA methylation at the core of this process. Current studies strongly predict that a hyper-methylated fibroblast phenotype promotes myofibroblast differentiation; however, the multiple gene-specific methylation events that stimulate this change in phenotype are not yet fully understood. Although there is significant evidence that epigenetic alterations compose a huge role in IPF pathogenesis, the factors that regulate these epigenetic alterations are not fully developed yet [97]. Application of what is previously known in the widely studied field of cancer biology may be enhanced to uncover specific epigenetic mechanisms that promote IPF. Since there are several similar aspects between these two pathologies, one common feature is the potent cytokine TGFβ and its augmented cell signaling through the SMAD family of proteins [98]. In some types of cancers, different levels of TGFβ expression are correlated with tumorigenesis, including abnormal cell proliferation and apoptotic events. Also, in cancer studies, TGFβ has been implicated with alterations in the epigenetic profile of cells. For example, in prostate cancer cells, TGFβ1 was shown to regulate expression of DNMT1. However, the blockade of TGFβ signaling decreased expression of all three DNMTs via a p-ERK-mediated pathway [99]. Further evidence of TGFβ regulation of epigenetic changes is shown by a study linking constant TGFβ signaling with increased DNA methylation coinciding with suppression of miR-200. This is also accompanied with sustained zinc finger E-box-binding homeobox (ZEB) expression [100]. Various studies emphasize the importance of this for transitioning between epithelial and mesenchymal phenotypic states, a key feature in lung fibrosis. It is astonishing that such familiar epigenetic responsive pathways can be active in all tissues studied, and that targeting them may help reduce myofibroblast differentiation and collagen deposition in any organ tested. Therefore, investigating epigenetic pathways and a clear understanding of the various mechanisms involved may contribute to an effective approach of epigenetic-altering therapeutics as anti-fibrotic agents [96,97].

### 3.6. Remodeling/Matrix

The ultimate result of IPF is abnormal tissue remodeling, characterized by extensive deposition of connective tissue followed by constant destruction of the lung parenchyma to form fibrotic lesions [94]. The molecular mechanisms responsible for abnormal matrix remodeling are poorly understood, although dysregulation in tissue turnover involves two families of proteins: matrix metalloproteinases (MMPs), and the tissue inhibitors of metalloproteinases (TIMPs). Recent evidence suggests that specific changes in both MMP and TIMP expression level and localization occur in the IPF lung microenvironment, revealing an upregulation of collagenase-1 (MMP-1) and gelatinases (MMP-2 and MMP-9), as well as the four TIMPs. Function depends on location; MMP-1 is mostly seen in alveolar macrophages and epithelial cells, but less seen in fibroblasts or the extracellular matrix (ECM) [101]. However, this epithelial location of MMP1 raises questions regarding its specific function, relatively far away from where fibrillar collagens are being deposited. One possible explanation could be related to alveolar epithelial cell migration, as projected for keratinocytes in skin wound healing. Thus, MMP1 is invariably expressed by basal keratinocytes in all forms of cutaneous wounds. In contrast, the TIMPs are highly expressed throughout the injured IPF lung. Specifically, TIMP-2 is mainly expressed by myofibroblasts within the fibroblastic foci. Therefore, it has been expected that a non-degradative lung environment dominates in IPF, which augments the continuous accumulation of fibrillar collagens. Moreover, fibroblasts derived from IPF lungs express in vitro higher amounts of TIMPs when compared to fibroblasts from normal lungs, without differences in MMP1 expression [12]. In addition, myofibroblasts represent an aggressive phenotype, being the main subset responsible for fibrillar collagen accumulation. In the case of gelatinase A and B, overexpression may result in a different situation. These MMPs have been confined, in sub-epithelial fibroblasts/myofibroblasts, occasionally in regions of exposed alveolar basement membranes, proposing that they might participate in basement membrane interruption. This pathological event may contribute to the failure of a successful re-epithelialization, but also promotes the migration of fibroblasts/myofibroblasts into the alveolar spaces [88]. Predominately, various studies indicate that epithelial cells are important in the initiation and progression of the disease. Thus, abnormally activated alveolar/bronchiolar cells release specific growth factors and cytokines responsible for proliferation/migration of local fibroblasts (e.g., platelet derived growth factor (PDGF)), as well as its transition to myofibroblasts. This usually occurs through the secretion of transforming growth factor beta-1 (TGFβ1) [76,102]. Integrin-αvβ6, which is capable of activating TGF-β and is expressed at low levels in normal lung tissue, is strongly upregulated in IPF. Also, epithelial cells contribute to the expansion of the fibroblast population through the epithelial to mesenchymal transition (EMT).Transforming an epithelial cell into a mesenchymal cell demands a profound change in genetic/epigenetic responses leading to variation in morphology, migration capacity, and cellular architecture [103]. Furthermore, various studies indicate that tumor-associated matrix metalloproteases (MMPs) can activate processes associated with EMT. Other MMPs, such as MMP-2 and MMP-9, mediated E-cadherin disruption as the main step in EMT indicating that it may be also implicated in the fibrotic response [14]. ECM alterations can also directly lead to inflammation. Components of the ECM, mainly elastin and hyaluronan, are reported to express chemotactic activity on inflammatory cells, induce immune responses, and enhance adhesion of polymorphonuclear neutrophils (PMNs), and alter gene expression profiles in inflammatory cells [85]. In wound repair, the glycosaminoglycan hyaluronic acid is abundant in the initial inflammatory phase, and acts as a promoter of early inflammation. However, failure to eliminate ECM degradation products from tissue injury sites may contribute to the unresolved inflammation and ongoing destruction observed in IPF. Clearance of hyaluronan fragments is thought to be dependent both on its receptor CD44 and on recognition by the host via toll-like receptor, TLR2 and TLR4. Hyaluronan-TLR2 and hyaluronan-TLR4 signals adjust both the epithelial cell integrity, as well as innate inflammatory response, which is important for recovery from acute pulmonary injury [104]. In addition, ECM has a critical role in leukocyte adhesion. For instance, adhesion of PMNs to ECM proteins has been reported to be critical for their migration, and accounts for PMN recruitment to sites of inflammation. PMN migration through the parenchyma can be affected by the ECM composition, with multiple studies demonstrating increased adherence of PMNs to surfaces covered with fibronectin and collagen. ECM components such as fibronectin can perform as chemotactic agents for PMNs, predicting that local rates of migration may be regulated by various ECM components. Therefore, a better understanding of the diversity of MMP proteolysis and matrix remodeling will help to define the fibrosis-specific responses of MMPs in IPF [14,88,105].

## 4. Conclusions

The wound-healing process involves injury followed by the inflammatory phase and subsequently, the proliferative phase. There are several types of clinical injuries to the human lung such as infection, trauma, and immune-mediated. These may be treated with antimicrobials, ventilator support, and anti-inflammatory agents. Lung wound normally heals without scarring and function is maintained. On the other hand, chronic nonhealing lung wounds such as those with usual interstitial pneumonia (UIP) can progress to end-stage lung disease and respiratory failure. UIP may occur following a known injury, such as with hypersensitivity pneumonitis or occupational/environmental lung disease. Full recovery is possible with removal of the instigating factor; however, in some cases, despite removal of the cause, dysfunctional wound healing occurs and disease progresses to the end stage. Current problems include the inability to identify and avoid the initial injury as in idiopathic disease. We are unable to predict when injury and treatment will heal appropriately or will heal dysfunctionally and lead to progressive UIP. There have been some promising discoveries in pharmaceutical management with drugs such as pirfenidone and nintedanib, which can slow the fibrotic process; however, we still do not have the ability to cure these diseases.

With better understanding of the factors that differentiate appropriate from dysfunctional wound healing, we may ultimately be able to intervene and prevent or halt the development of inappropriate fibroblastic activity in the lung and avoid the development of end-stage lung disease.

## Figures and Tables

**Table 1:** Prevalence and incidence of IPF by geographic district [22,23].

Country and Study Period First Author (Ref.)	IPF Prevalence/100,000 Population			IPF Incidence/100,000 Population			Case Detection and Study Method
	All	Male	Female	All	Male	Female	
USA							
1988–1990 Coultas et al. [26]		20.2	13.2		10.7	7.4	Population-based
1997–2005 Fernandez Perez et al. [27]	27.9–63.0						ILD registry Population-based medical record linkage system (2000 ATS/ERS criteria)
Europe							
Czech Republic 1981–1990 Kolek et al. [28]	12.1			0.94			Clinical registry
Finland 1997–1998 Hodgson et al. [29]	16–18						Pulmonary clinic database
Greece 2004 Karakatsani et al. [30]	3.38			0.93			National survey of pulmonologists (2000 ATS/ERS criteria)
UK 1991–2003 Gribbin et al. [31]				4.6	5.69	3.44	National-wide primary care database
UK 2000–2009 Navaratnam et al. [32]				7.44	9.46		National-wide primary care database
Asia							
Taiwan 1997–2007 Lai et al. [33]	0.7–6.4			0.6–1.4			Taiwanese national health insurance system
Japan 2005 Ohno et al.[34]	2.9						Medical benefits records
